# Protocol for the End-of-Life Social Action Study (ELSA): a randomised wait-list controlled trial and embedded qualitative case study evaluation assessing the causal impact of social action befriending services on end of life experience

**DOI:** 10.1186/s12904-016-0134-3

**Published:** 2016-07-13

**Authors:** Catherine Walshe, Guillermo Perez Algorta, Steven Dodd, Matthew Hill, Nick Ockenden, Sheila Payne, Nancy Preston

**Affiliations:** International Observatory on End of Life Care, Division of Health Research, Lancaster University, Bailrigg, Lancaster LA1 4YW UK; Institute for Volunteering Research, NCVO, Society Building, 8 All Saints Street, London, N1 9RL UK; Division of Health Research, Lancaster University, Bailrigg, Lancaster LA1 4YW UK

**Keywords:** Palliative care, Public health, Social action, Quality of life, Trial

## Abstract

**Background:**

Compassionate support at the end of life should not be the responsibility of health and social care professionals alone and requires a response from the wider community. Volunteers, as community members, are a critical part of many end-of-life care services. The impact of their services on important outcomes such as quality of life is currently poorly understood. The purpose of this study is to evaluate a series of social action initiatives which use volunteers to deliver befriending services to people anticipated to be in their last year of life. The aim is to determine if receiving care from a social action volunteer befriending service plus usual care significantly improves quality of life in the last year of life.

**Methods/design:**

The research questions will be addressed through a wait-list randomised controlled trial (WLRCT) and qualitative case study evaluation across 12 sites in England. Participants will be randomly allocated to either receive the social action volunteer befriending service straight away or receive the intervention after a four week wait (wait-list arm). The impact of the intervention on end-of-life experience (quality of life as primary outcome, loneliness, social support) will be measured. Repeated assessments will be carried out at baseline and weeks 4 and 8 for the intervention arm and weeks 4, 8 and 12 for the wait-list arm. For selected sites case study evaluation will include interviews, observation and documentary analysis to understand the mechanisms underpinning any found impact.

**Discussion:**

This study will address the need to both provide services which use social action models to support end-of-life care in community settings, and to robustly evaluate these models to determine if they influence the experience of end-of-life care. Such services could work to reduce isolation, help meet emotional needs and maintain a sense of connectedness to the community. ISRCTN 12929812 Registered 20.5.15

## Background

Providing excellent end-of-life care (defined here as care in the last year of life) that is responsive to need is critically important. People want to be cared for as close to home as possible, especially in the last year of life [1]. However there is poor understanding about which components of home based interventions provide the highest benefit [2, 3]. Understanding the impact of these components in context is important so that priority is given to developing services in a way known to maximise benefit to people in their last year of life, informal carers and the services themselves.

With reference to end of life care at home, it is increasingly recognised that compassionate support cannot be the responsibility of health and social care professionals alone and requires a response from the wider community. Volunteers are a critical part of many end-of-life care services [4–11] Family members are satisfied with the services that volunteers provide at the end-of-life [9], but there is little evidence of their effect on care outcomes. A recent systematic review assessing the impact of volunteers involved in the direct care of palliative care patients and their families at the end-of-life only found 8 studies, none from the UK. They indicate that volunteer involvement has a positive impact on satisfaction with care and that people may survive longer with home visits from a volunteer. They conclude that further research is needed to ensure the resource of volunteers in palliative care is used appropriately and effectively. Evaluation in well-designed comparative studies is recommended [12].

In order to meet this knowledge gap, the UK Cabinet Office funded the provision and evaluation of a range of social action projects at the end of life. The purpose of this evaluation is to use a robust and ethical comparative design to determine the effect of volunteer led social action befriending services on quality of life in the last year of life and how such services can best be implemented and delivered. Research of this sort can ensure that the resource of volunteers in palliative care is used appropriately, and in a way which effectively improves quality of life and the experience in the last year of life. Many volunteer delivered befriending social action services are being run or developed to provide non-clinical support to people in their own homes such as companionship, running errands and providing information. Whilst this feels intuitively helpful, there is no robust evaluation of the outcomes of these services nor understanding of how best to provide such care to maximise effectiveness.

## Methods/design

### Aims

The **primary aim** of the study is to evaluate the effectiveness of receiving care from a social action volunteer befriending service plus standard care at improving quality of life as compared to usual care alone for adults in the last year of life.

The **secondary aims** are to:

- explore whether the social action volunteer befriending service reduces loneliness and affects the perception of social support for adults
- examine whether informal carers for those receiving care from a social action volunteer befriending service experience less carer burden
- determine whether receiving care from a social action volunteer befriending service can affect participant’s use of other health and social care services
- identify and explore the factors that influence the impact of social action volunteer befriending services on end-of-life experience

### The intervention

The intervention is based on a model of social action, and comprises a number of components. Core to the intervention is that it is provided by a trained volunteer rather than a paid member of staff, and provides non-clinical, non-hands on support. Individual support needs are assessed by the non-clinical volunteer co-ordinator, and support then provided from a suite of possible options including ‘befriending’ e.g. sitting with someone to provide companionship, ‘practical support’ e.g. assisting with household tasks such as dog walking, gardening, picking up prescriptions or other errands, and ‘signposting’ e.g. providing information on other available services. Support will be typically provided from 1–3 times per week, face to face or by telephone, as negotiated between volunteer providers and participants. Participants will be in the trial for 8 or 12 weeks (immediate vs wait-list arm), but their receipt of the social action volunteer service can continue outside the trial beyond this point if needed. Participants are free to withdraw at any time. Participants will continue to receive all usual care during this period. In this study the intervention will be provided across a number of sites in England who are independently funded to provide this service by the UK Cabinet Office.

### Methods

The study involves two main approaches to data collection.

#### Wait-list randomised controlled trial

This is a multi-site (*n* = 12) randomised controlled trial (RCT) involving a wait-list design. Participants will be allocated on a 1:1 allocation ratio. A wait-list trial was chosen as an ethical design which has the strength of an RCT, but with greater acceptability as all patients eventually receive the intervention. In a wait-list trial, respondents are either allocated to an intervention group where they receive the intervention immediately (or as soon after randomisation as possible) or to a wait-list group where they receive the same intervention after a defined period on a waiting list. While they are on the waiting list patients receive standard/usual care. Wait-list designs have been successfully used in end-of-life care research, including where there is a pre-existing service [13, 14]. Advantages include having the strength of a RCT in determining causality, but with greater acceptability as all patients eventually receive the intervention which can feel more ethically defensible in end of life care [13–18]. We propose a short wait period to minimise risk and acknowledge that interventions need to be effective in a short time period where life expectancy is short (i.e. a year or less) and attrition due to illness or death highly anticipated [19].

#### Qualitative case study design

Results generated from the WLRCT will be combined with a qualitative case study evaluation to understand the impact of social action befriending services on end-of-life experience and the factors that can maximize or minimise that impact. Each site providing the service comprises a case. The qualitative work stream (case study evaluation) comprises three distinct research activities within each chosen case study site:

- qualitative interviews with i) Patient participants and informal carers both receiving and having waited to receive the intervention to explore their experience of the service (*n* = 3-6); ii) volunteer staff providing the intervention to explore their experience of providing care, motivations, training, and the research study design etc. (*n* = 3-6); and iii) staff e.g. social action volunteers key manager/coordinator, other responsible stakeholders e.g. chief executive or general manager, clinical care staff etc. to explore organizational culture, history of the programme, selection, training and support of volunteer/ social action team. Interviews will last approximately an hour and will be conducted either face to face or via phone/skype. All participants taking part in the interviews must be able and willing to give informed consent.
- Non participant observation of relevant organizational meetings, workload allocation, decision-making activities etc.
- Documentary data such as service policies, job descriptions and other relevant written materials about the services provided as well as contextual data from the end-of-life care intelligence network on issues such as place of death etc.

## Study setting

Twelve individual sites across England will participate in this study. Six are run by the same national organisation. Ten of the sites are hospices, providing end-of-life care to their populations, one site provides a service jointly organised between local hospices and a NHS Trust, and one site is a charity providing care and services to people with substance abuse issues.

### Eligibility for sites to join the study

Sites that will participate in this study have been selected as part of a government funded initiative to support social action at the end-of-life in England. A number of organisations tendered to provide these services against a set specification, and were then shortlisted for interview. Selection considerations at shortlisting and interview included the sites’ match to the tender, their capacity to deliver the proposed service, and their ability and willingness to contribute to the evaluation of these services. All sites were aware at interview that they would be providing their services in the context of this study design (Wait-list trial and case study evaluation).

## Study population

### Inclusion and exclusion criteria

#### Patient participant inclusion and exclusion criteria

Outside a trial context, it is anticipated that a wide variety of people could be referred to such services with a range of prognosis, diagnosis and backgrounds. We therefore propose the widest possible inclusion criteria to ensure that as many people as possible are eligible (to match likely future service referral criteria).

##### Inclusion criteria

1. Those eligible to be referred to an end of life care service determined by the referring organisation/individual. They should be able to answer ‘no’ to the ‘surprise question’: ‘Would you be surprised if the patient dies within a year?’
2. Able to give informed consent.

##### Exclusion criteria

1. Age <18 years
2. Those who only understand or speak a language in which our main outcome measure (the WHOQOL-BREF) is unavailable. This is anticipated to be a very small number of potential participants as the WHOQOL-BREF is available in a wide range of languages, including the main languages spoken in the participating sites.
3. Those with an anticipated prognosis of < 4 weeks

#### Family/informal carer

At inclusion, patient participants will be asked to also identify a family member/informal carer to participate in the study. Carers, who may or may not be family members, will be defined as lay people in a close supportive role who share in the illness experience of the patient [20] or provide emotional support. Patients who are unable to identify a family member or informal carer at inclusion will not be excluded from the study.

### Qualitative case study eligibility criteria

The patient participant inclusion criteria for those invited to participate in the qualitative component of the study are the same as for the trial component. Family members/informal carer and those involved in managing and providing the intervention (e.g. volunteers, service managers, health and social care professionals etc.) will be also invited to participate in interviews.

Informal/family carer inclusion criteria for qualitative case study interviews:

1. Identified as a family/informal carer by the patient participating in the trial/qualitative case study
2. Over 18 years
3. Able to give informed consent at the time of the interview

Volunteer/staff inclusion criteria for qualitative case study interviews:

1. Involved in provision or management of the service providing the social action befriending service at the chosen case study site.

### Study sample size

#### Trial study size

Sites funded to provide these services provided an estimate of likely referral numbers to their services. These aggregated numbers indicate that 700 participants are likely to be referred to the service in the specified trial time period which indicates a maximum potential study size of 350 patients in the intervention arm and 350 patients in the wait-list arm of the study recruited from the 12 sites across England involved in the study. Given that each eligible patient will be asked to nominate a carer, the total number of carers to be recruited will be up to 700.

#### Qualitative study size

Three to six (patients, informal carers and volunteers) and two to three (key managers or co-ordinators) will be invited to an interview per case study site. Eight sites will be selected according to their geography and provider type from the 12 sites involved in the study comprising a total sample size of 88–168 participants.

### Power calculation and sample size

#### Trial power calculation and sample size

Three hundred fifty patients per arm are considered to be sufficient to examine the impact of social action services (intervention) on quality of life (primary outcome). Trial power was estimated using a worst case scenario assuming 5 % attrition at primary outcome measure. With 350 or more participants per arm power will exceed .80 to detect difference in change over time corresponding to an effect size of f = .10 (considered a small effect size) between the intervention and wait-list groups. This power model uses alpha = .05, two tailed, and uses a conservative correlation of *r* = .6 for scores lagged 4 weeks, and *r* = .5 for 12 weeks.

#### Case study sample size

A sample size of 8 organisational case studies is considered to be sufficient to understand the processes and determinants that can potentially maximize or minimise the impact of social action services (intervention) on end of life experience. Cases will be sampled from individual study sites chosen on the basis of their geography in order to i) maintain a balance between the South and North of England and ii) maximise heterogeneity on the basis of the type of the service provider (e.g. hospice, social care or voluntary sector organisation).

### Study procedures

The risk of sites experiencing challenges with study procedures will be minimised by providing study sites with clear information with what the study involves, providing access to members of the research team, to answer queries and address problems experienced by each study site. Participants’ engagement with the intervention will be facilitated via a detailed participant information sheet explaining the nature and the particulars of the study that will be handed out to all participants prior to commencing the study.

#### Trial recruitment and informed consent procedure

All those referred to the participating services will be invited to take part in the study. Services will be provided with information to give to potential referrers so that they understand the trial component of service delivery, and the trial eligibility criteria. Appropriately trained staff at the sites receiving referrals (with Good Clinical Practice and study specific training on trial and consent procedures) will assess all referrals for eligibility for the study against trial criteria. Those who are eligible will be given an invitation letter and participant information sheet and given time to ask questions and consider trial participation. Once a referred patient has indicated that they are happy to participate in the trial, the informed consent documentation will be explained to them and written consent taken. These staff are also responsible for informing Lancaster University of ay adverse events. Baseline data will then be collected. At this point the site will contact Lancaster University as part of the randomisation procedure, and plan intervention delivery according to the assigned trial arm.

For trial participants who are receiving their service within a chosen case study site, a proportion of them may be invited to participate in a qualitative interview to discuss their experience of the service. Participants will give consent for this at initial recruitment and again give written consent prior to interview.

#### Randomisation of patients in the trial

Once written informed consent has been obtained to participate in the trial, and baseline data collected, patients will be randomly allocated (1:1 allocation ratio) to either the intervention or the wait-list arm of the trial. Site coordinators will contact a randomisation line at Lancaster University, and the next sequence in the allocation (stored in sequentially numbered sealed opaque envelopes) will be revealed. The randomisation sequence will be computer generated by an experienced statistician outside the study context, with rebalance in the arms after 10 randomisations. Blinding of site staff and patient participants will not be possible due to the nature of the intervention.

## Data collection methods

### Outcome measures

The causal impact of the intervention on each aspect of end of life experience examined in this study will be measured using a pre-determined set of outcome measurement tools as follows:

- Quality of life (primary outcome measure) is measured using the World Health Organisation Quality of Life (WHOQOL-BREF) Scale, a short validated measure of quality of life and wellbeing, having wide breadth. Our primary outcome will be overall quality of life (single response question), with secondary outcomes the quality of life domains measured by the WHOQO-BREF (social, environmental, psychological and physical domains) [21].
- Loneliness (secondary outcome measure) is measured using the De John Greiveld 6-item Loneliness Scale, a short, well-used, reliable and valid measurement instrument for overall, emotional, and social loneliness, chosen for brevity and relevance of the items when mapped onto anticipated outcomes [22].
- Social Support (secondary outcome measure) is measured using the 8-item modified Medical Outcomes Study Social Support Survey (mMOS-SS), a short validated scale covering two domains (emotional and instrumental social support) designed to identify potentially modifiable social support deficits, chosen for brevity and relevance of the items when mapped onto anticipated outcomes [23].
- Carer Burden (secondary outcome measure) is measured using the Caregiver Burden Scale-End of Life Care (CBS-EOLC), a reliable and valid measurement tool designed to specifically assess family caregivers’ burden within the palliative care context, chosen for brevity and relevance of the items when mapped onto anticipated outcomes [24].

Socio-demographic data (age, gender, disease diagnosis, education, marital status, living status, spirituality and ethnicity) in the form of a self-completed questionnaire will be collected from both patients and informal carers at baseline. At baseline and subsequent time points patient participants will also be asked to indicate the number, type and frequency of contact they have with networks of others (to include social networks and contact with health and social care service providers). After baseline, week 4, 8 (and 12) questionnaires (with paid return envelope) will be mailed to participants by research staff at Lancaster University, with reminders after 2 weeks if required.

### Participant timeline

#### Patient questionnaires

Baseline data (including socio-demographic data such as age, gender, disease diagnosis, education, marital status, living status, spirituality and ethnicity) will be collected from both patients and carers prior to randomisation. The patients randomised to immediately receive the service will do so (following any service administrative procedures) and continue to receive it for as long as required as assessed by the service providers in conjunction with service recipients. At week 4 all participants (intervention arm and wait-list arm) will complete the outcome measures. At this point, the patients on the wait-list arm will also commence receiving the service. Measures will be repeated at week 8. those on the wait-list arm will complete an additional questionnaire at week 12. Participants who withdraw from the service will not be required to complete questionnaires as this may be due to deterioration in physical condition, and the risk of sending questionnaires inappropriately if the services are unable to provide updates on their health status.

#### Family members/Informal carers questionnaires

Patients’, family members/ informal carer complete socio-demographic variables and an outcome measurement tool for carers (CBS-EOLC). Repeated assessment will be carried out using the same tools (time points).

### Additional data

Each participating site will collect information about volunteer visits and record per patient participant details such as: referral date, consent date, allocation arm, dates of assessments date allocated to a volunteer, first date of volunteer visit, dates/numbers of volunteer visits and brief indication of support given and dates/numbers of volunteer support through calls/texts or skype.

A schedule of enrolment, interventions and assessment of outcomes is provided in Table 1.

**Table 1:**
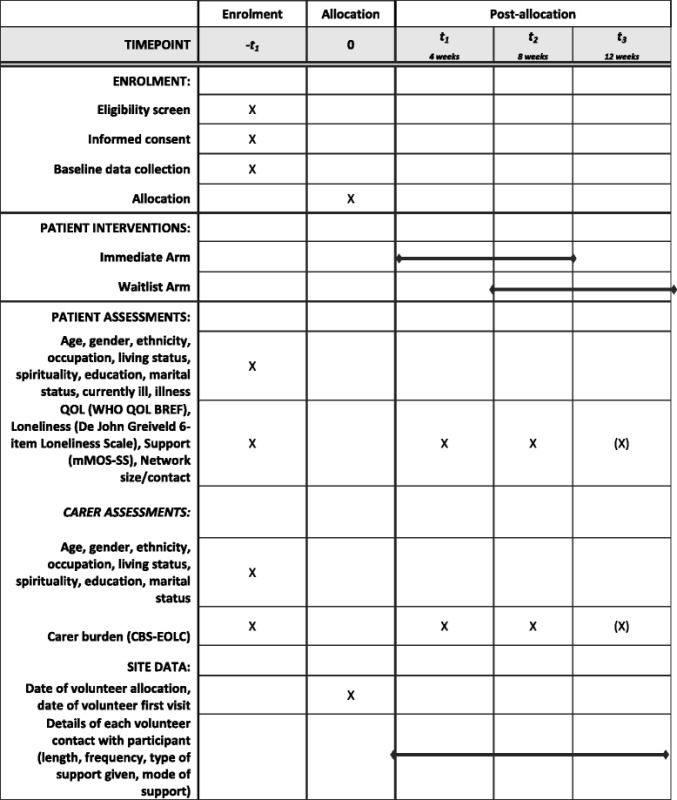
ELSA: Schedule of enrolment, interventions, and assessments

(X) indicates that week 12 data are only collected for those in the wait arm of the trial (8 weeks after commencement of intervention)

## Data analysis

### Descriptive analyses

Exploratory data techniques will be used to examine all distributions of outcome variables Continuous data will be summarised using means and standard deviations (SD) if normally distributed and medians and interquartile ranges (IOR) if non-normally distributed. Categorical data will be described using frequencies and percentages.

### Comparison between arms

Data will be analysed on an intention to treat basis. A linear mixed effect model (LME) will be fitted to each outcome variable at 4, 8 and 12 weeks. The random effects are intercept and slope; which posit that each participant has a “personalized” linear response to treatment. The fixed effects include site, treatment group, and time since entry, and time interactions, which measure systematic differences in the rate of change. The assumption of a common initial mean for all participants is justified by the randomization of participants into treatment groups. A major advantage of the LME model is that is does not require a balanced design; in particular, participant effects can be estimated using incomplete data. However, missing-completely-at-random (MCAR) assumption will be tested, and in case that this assumption is not tenable, multiple imputation procedures will be employed.

Analyses will be conducted to evaluate potential variables associated with dropout in wait-list controls, to prevent that this attrition inflates the apparent treatment effect at 4 weeks by comparing the intervention group with only those in the wait-list group with some particular features. If observed, these confounders will be included in the LME model to reduce the bias of differential attrition on the estimated treatment effect.

Statistical significance will be assessed at the 5 % (two-sided) level. All statistical analyses will be conducted using SPSS v.20.

### Missing data

A complete case analysis will be undertaken with a range of approaches for undertaking sensitivity analyses to access the robustness of the findings with respect to missing data.

### Case-study evaluation analyses

Data analyses will follow a framework analysis approach using a matrix approach informed by the final theory of change. Framework analysis facilitates within and cross case pattern matching and has been used in case studies in palliative and end-of-life care [25, 26]. This analysis will be integrated with trial data so that an understanding of the factors affecting impact can be compared with quantitative impact. Cross case pattern matching will follow to identify thematic factors associated with challenges and successes in creating impact. This should provide information on facilitators, challenges, barriers and strategies for overcoming them.

All qualitative analyses will be performed using NVivo software.

## Handling and storage of data and documents

### Access to data

The designated contact person(s) in each study site will have access to clinical information either contained within the service referral or available within the organisation if the potential participant is known to them already. Patient and carer permission will be required for Lancaster University to access patient contact details in order to post subsequent questionnaires to patient and carer participants. Patient identifiable data will be encrypted and securely transferred. To ensure confidentiality, data dispersed to project team members will have any identifying participant information removed.

Study data (baseline, week 4, 8, 12 questionnaires) will be returned by post to Lancaster University for entry onto an SPSS database. Weekly data on volunteer input will be securely transferred electronically to Lancaster University for entry onto SPSS. Data will be cleaned and quality checked independently from the person entering the data. Range checks will be made for expected data values. An error rate will be reported.

### Confidentiality

Questionnaires will be coded with a unique alpha numeric identifier which will be kept separately from participant contact details. No personal identifying information will be included in the questionnaires. Interviews will be recorded only if the patient consents for this to occur. Interviews will be digitally recorded on an encrypted recorder and transferred as soon as possible to a password protected computer.

### Data storage

Data storage and handling will include locked storage, password protection, encryption and anonymisation of original data. All audio − recordings will be labelled only with participants’ alphanumeric codes and stored securely. Direct quotations from respondents will only be used in such a way as to ensure anonymity.

### Data monitoring

Due to the low risk nature of the intervention, the REC determined that a data monitoring committee (DMC) was not required. Regular meetings of protocol authors and funder representatives will occur where any adverse or unintended effects of trial interventions or conduct will be discussed, and stopping decisions made. Site initiation visits will be made by Lancaster University investigators to explain trial conduct, and an interim audit of site trial procedures will be made by the investigators.

## Discussion

### Risks

The main risk identified is that participants entered into the trial who are randomised to the wait-list condition will potentially receive suboptimal support over that waiting time, if the intervention is found to be of benefit. However a number of factors mitigate against this risk:

- First, there is insufficient current evidence that such services are of benefit. There are no current trials or comparative studies of such social action services which measure outcomes and recent reviews of the literature about volunteering at the end-of-life argue strongly for well conducted robust evaluations of these services.
- Second, a relatively short wait period has been chosen to minimise any risks should the service prove to be of benefit. In the UK people are used to short waits for services and are unlikely to feel seriously disadvantaged by a short wait.
- Third, these social action services are not currently available to participants, and they would not be receiving the potential benefit of these services without the funding available to support this trial.

There is a further risk that these services cause burden, distress or harm rather than the intended benefit. This is unlikely to be the case, as other similar services have had reports of satisfaction, and the service provided is not core clinical work or hands on care but additional social support. The qualitative interview data collected from participants should give an indication of experience which can be used to monitor this possibility. Participants will also have written information given to them, and verbally explained, about the complaints and other procedures to follow if they have any concerns or feedback related to either their service provision or the conduct of the research. The research team and service provider sites will work closely together to monitor any issues and act upon them rapidly.

There is also a risk that patient and carer participants may be concerned or distressed by some of the questions asked within the questionnaire toolkit. This risk will be minimised by using existing well validated standardised tools, which have been tested in a number of similar populations, and in different cultures and contexts. The tools have been carefully chosen to be short and easy to complete to minimise participant burden as much as possible.

Finally, the risk that participants will be contacted to complete subsequent questionnaires or to be invited to interview when they may be too unwell through deterioration in their condition, or that carers may be distressed by such information after a participants’ death will be mitigated by a clear protocol between the sites and the University to ensure that this information is securely transferred to avoid such distress.

### Potential benefits

Whilst no direct benefit to participants has been promised, they may enjoy and benefit from the social action intervention. This intervention can continue after the research phase of the service: services have been asked to provide this service as per their assessment of need, the only difference to usual service provision is the wait period for those allocated to the wait-list condition. Indirectly, our experience of conducting research in palliative care has shown that patients and informal carers have been willing to take part in such studies as a method of reciprocity for the care they have received from palliative care practitioners and in the knowledge that their participation will inform the overall understanding of the provision of care and benefit others in the future; this finding is increasingly being supported by a growing literature [27–29]. In addition, experience with this kind of research shows that patients and carers often appreciate the interest shown in their experience and opinions [30].
