# Erratum to: Protocol for the End-of-Life Social Action Study (ELSA): a randomised wait-list controlled trial and embedded qualitative case study evaluation assessing the causal impact of social action befriending services on end of life experience

**DOI:** 10.1186/s12904-016-0170-z

**Published:** 2016-12-29

**Authors:** Catherine Walshe, Guillermo Perez Algorta, Steven Dodd, Evangelia Papavasiliou, Matthew Hill, Nick Ockenden, Sheila Payne, Nancy Preston

**Affiliations:** 1 International Observatory on End of Life Care, Division of Health Research, Lancaster University, Bailrigg, Lancaster LA1 4YW UK; 2 Institute for Volunteering Research, NCVO, Society Building, 8 All Saints Street, London, N1 9RL UK; 3 Division of Health Research, Lancaster University, Bailrigg, Lancaster LA1 4YW UK

## Erratum

Evangelia Papavasiliou worked on this protocol and the first few months of the project, but moved before the submission of the paper and was erroneously omitted in the original version of the paper [1]. The authors would like to acknowledge her work on the protocol and as a contributing author, and her details are therefore included in this erratum.

With this in mind, the authors would also like to update the sections mentioned below:

New ‘Competing Interests’ statement:

The individuals (principal investigators and research associates) and the institutions involved in this trial declare no conflict of interests.

New ‘Author Contributions’ statement:

The study was conceived by CW, NP, SP, GPA and NO. SD, EP and MH are responsible for operationalising the protocol. CW is chief investigator for the study.

New ‘Acknowledgement’ Section:

We acknowledge the contributions of the people who take part in this research, often at a time of great challenge in their lives, thank you. This study would not be possible without the support of the participating sites, who are responsible for identifying participants, taking consent, and managing study participants and site specific documentation.
